# A novel defined TLR3 agonist as an effective vaccine adjuvant

**DOI:** 10.3389/fimmu.2023.1075291

**Published:** 2023-01-24

**Authors:** Kwang Hyun Ko, Seung Bin Cha, Seung-Hwan Lee, Hyun Shik Bae, Chul Soo Ham, Min-Gyu Lee, Dong-Ho Kim, Seung Hyun Han

**Affiliations:** ^1^ Research and Development Center, NA Vaccine Institute, Seoul, Republic of Korea; ^2^ Interdisciplinary Program in Genetic Engineering, College of Natural Sciences, Seoul National University, Seoul, Republic of Korea; ^3^ Department of Oral Microbiology and Immunology, and Dental Research Institute, School of Dentistry, Seoul National University, Seoul, Republic of Korea

**Keywords:** vaccine adjuvant, dsRNA, *in vitro* transcription, TLR3 agonist, Th1 response

## Abstract

Synthetic double-stranded RNA analogs recognized by Toll-like receptor 3 (TLR3) are an attractive adjuvant candidate for vaccines, especially against intracellular pathogens or tumors, because of their ability to enhance T cell and antibody responses. Although poly(I:C) is a representative dsRNA with potent adjuvanticity, its clinical application has been limited due to heterogeneous molecular size, inconsistent activity, poor stability, and toxicity. To overcome these limitations, we developed a novel dsRNA-based TLR3 agonist named NexaVant (NVT) by using PCR-coupled bidirectional *in vitro* transcription. Agarose gel electrophoresis and reverse phase-HPLC analysis demonstrated that NVT is a single 275-kDa homogeneous molecule. NVT appears to be stable since its appearance, concentration, and molecular size were unaffected under 6 months of accelerated storage conditions. Moreover, preclinical evaluation of toxicity under good laboratory practices showed that NVT is a safe substance without any signs of serious toxicity. NVT stimulated TLR3 and increased the expression of viral nucleic acid sensors TLR3, MDA-5, and RIG-1. When intramuscularly injected into C57BL/6 mice, ovalbumin (OVA) plus NVT highly increased the migration of dendritic cells (DCs), macrophages, and neutrophils into inguinal lymph node (iLN) compared with OVA alone. In addition, NVT substantially induced the phenotypic markers of DC maturation and activation including MHC-II, CD40, CD80, and CD86 together with IFN-β production. Furthermore, NVT exhibited an appropriate adjuvanticity because it elevated OVA-specific IgG, in particular, higher levels of IgG2c (Th1-type) but lower IgG1 (Th2-type). Concomitantly, NVT increased the levels of Th1-type T cells such as IFN-γ^+^CD4^+^ and IFN-γ^+^CD8^+^ cells in response to OVA stimulation. Collectively, we suggest that NVT with appropriate safety and effectiveness is a novel and promising adjuvant for vaccines, especially those requiring T cell mediated immunity such as viral and cancer vaccines.

## Introduction

Vaccine adjuvants are substances that improve the efficacy of vaccines by helping induce strong protective immune responses to vaccine antigens in humans or animals (1). Adjuvants can be used to (i) maximize the immune responses to vaccines, (ii) guide the type of adaptive immunity specific for each pathogen type, or (iii) alter the speed, generation, breadth, specificity, and affinity of the immune responses (2). So far, multiple mechanisms of action for adjuvants have been elucidated (3). One is the depot formation that holds and slowly releases antigens, prompting sustained stimulation of the immune system (4). Another is to promote the recruitment of the immune cells and maturation and activation of antigen-presenting cells (APCs) through the production of cytokines and chemokines (4). Mature APCs then generate an adaptive immune response with enhanced antigen processing and presentation capabilities (4). On the other hand, adjuvants can modulate the type of immunity, for example, dominating either cell-mediated immunity or humoral immunity by the induction of different types of cytokines (5). In fact, informed choice of vaccine adjuvant suitable for target antigens is essential for the promotion of the desired vaccine efficacy.

The first adjuvant used in human vaccines was aluminum salts (referred to as alum) such as aluminum hydroxide, aluminum phosphates, and aluminum potassium sulfate, mainly inducing robust Th2 and antibody responses (1, 6). Alum adjuvants have been widely used since 1930s for licensed vaccines such as diphtheria and tetanus vaccines targeting pathogens for which antibody responses protect the host (1). Therefore, alum adjuvants may not be a good choice for vaccines against intracellular pathogens which largely require the induction of cell-mediated immunity. Additionally, they often induce over activation of Th2-type immunity resulting in adverse effects including antibody-dependent enhancement of infection (7). These limitations have facilitated the development of new adjuvants. QS-21, a saponin compound extracted from the Chilean soapbark tree, increases both antigen-specific antibody and cellular responses (3). It has been used as an adjuvant in melanoma and prostate cancer vaccines (8), as well as combining AS01 for Shingrix (herpes zoster vaccine) and Mosquirix (malaria vaccine) (3, 9). MF59, an oil-in-water emulsion comprising squalene, has been used in influenza vaccines for decades because of strong T and B cell responses (9–11).

Recently, natural ligands or synthetic agents for pattern recognition receptors such as Toll-like receptors (TLRs) have been developed as novel adjuvants, either alone or in combination with various formulations. For example, TLR4 agonist monophosphoryl lipid A is a major component of AS04, an adjuvant for a licensed human papillomavirus vaccine (Cervarix) (6, 9). TLR9 agonist CpG 1018 was clinically applied as an effective adjuvant of the hepatitis B vaccine (Heplisav-B and Dynavax) eliciting a strong antigen-specific Th1 and cell-mediated immune response (12). Poly(I:C), a synthetic TLR3 agonist, has been tried for clinical application in anti-cancer vaccines because it can induce strong T cell-mediated immune responses (13, 14). However, its instability and toxicity have led to the generation of its derivatives (15). Currently, two analogs of poly(I:C) have been subjected to clinical trials (16). For instance, Ampligen (also known as polyI:C12U) induces type I IFN production and Th1 response with reduced toxicity compared to poly(I:C), and is under phase II clinical trials for vaccines against melanoma, colorectal cancer, and prostate cancer (16). The vaccine adjuvant Hiltonol (Poly-ICLC) has been reported to promote an anti-cancer immune response that lowers the risk of metastatic recurrence in breast cancer patients and four studies are under clinical phase I/II trials (16).

Although dsRNA including poly(I:C) are promising adjuvant candidates, especially for anti-viral or anti-cancer vaccines, because of enhancing cell-mediated immunity as well as humoral immunity, there are serious shortcomings to overcome. Firstly, poly(I:C) is inherently not homogenous due to technical limitations in the manufacturing process (17). For the development of pharmaceuticals, it is necessary to define each active ingredient, but there are difficulties in identifying the exact size, molecular weight, and structure of poly(I:C) due to its heterogeneous characteristics. Secondly, since the biological activity of dsRNAs such as poly(I:C) varies depending on their molecular size (18), heterogeneity of poly(I:C) may lead to inadequacies in pharmacokinetic tests required for the development of drugs such as anti-cancer agents (19). In fact, batch-to-batch variations have been reported in the pharmacological action of poly(I:C) (20). Thirdly, the heterogenic nature of poly(I:C) is associated with toxic effects (21). Many studies have reported that poly(I:C) could cause autoimmune diseases such as primary biliary cirrhosis and lupus nephritis due to non-specific immune responses and systemic allergic reactions (22, 23). Fourthly, the production process is too complicated and inefficient to go to quality-controlled mass production. Indeed, poly(I:C) is synthesized through enzymatic synthesis using polyribonucleotide nucleotidyltransferase, but complicated reactions using the enzyme make it difficult to control the length of poly-I and poly-C molecules (20). Commercially-available poly(I:C) and its derivatives are highly heterogenous and lead to lot-to-lot variation (24). Moreover, heating and slow cooling many times are required for reannealing the poly-I and poly-C strands, which makes its reconstitution difficult (15). Thus, an advanced TLR3-based adjuvant would be an excellent next-generation adjuvant to retain the adjuvanticity of poly(I:C) but overcome its limitations. In the current study, by using polymerase-chain reaction (PCR)-coupled bidirectional *in vitro* transcription (IVT), we developed a homogeneous and quality-controlled dsRNA-based TLR3 agonist as an effective vaccine adjuvant and named it NexaVant (NVT).

## Materials and methods

### Antibodies and animals

Antibodies used in this study were purchased from BioLegend (San Diego, CA, USA) or Southern Biotechnology (Birmingham, AL, USA), and listed in **Table S1**. Specific pathogen-free C57BL/6 or BALB/c female mice at 6 weeks of age were purchased from Samtako Bio Korea (Kyounggi, Korea) and maintained at the NA Vaccine Institute (NAVI) animal facility (Seoul, Korea). The mice were fed a sterile, commercial mouse diet and provided with water *ad libitum*. The experimental protocols used in this study were reviewed and approved by the Ethics Committee and Institutional Animal Care and Use Committee (Permit Number: NAVI-2019-0002) of the NAVI.

### Synthesis of double-stranded RNA and NVT

As a template for IVT, the nucleotide segment (1,701-3,360; 1,660 nucleotides) from CSBV genome (GenBank accession number KF960044.1) was cloned into the pUC-GW-Amp vector and the template was amplified by PCR using primer pairs containing the T7 RNA promoter sequence. PCR cycling conditions were as follows: 95°C for 5 min, followed by 35 cycles of 95°C for 30 s, 60°C for 30 s, and 72°C for 1 min. A final elongation step was carried out for 5 min at 72°C. Synthesis and purification of dsRNA were performed using a MEGAscript RNAi Kit (Thermo Fisher Scientific, Waltham, MA, USA) according to the manufacturer’s instructions. Briefly, IVT reactions were carried out by incubating the PCR products with 10× T7 Reaction Buffer, 4 ribonucleotide solutions, and T7 Enzyme Mix at 37°C for 2-4 h. The IVT products were incubated at 75°C for 5 min and then the complementary RNAs were allowed to anneal to each other at room temperature to form dsRNA. The template DNAs and unannealed ssRNAs were removed by treatment with DNase I and RNase A. Lastly, each dsRNA sample was purified with 100% ethanol and eluted with a solution containing 10 mM Tris-HCl (pH 7) and 1 mM EDTA. For the synthesis of NVT used as a vaccine adjuvant, partial nucleotide segment (1,701-2,112; 412 nucleotides) was selectively cloned, and ssRNAs were digested by treatment with RNase T1 instead of RNase A. Template and primer details for NVT synthesis are shown in **Table S2**.

### Reversed phase-high performance liquid chromatography

The purified NVT sample was analyzed by RP-HPLC on the Waters Alliance HPLC System (Waters, Milford, MA, USA) using a Waters XBridge OST C18 Column with 130 Å, 2.5 μm, and 4.6 × 50 mm (Waters, Milford, MA, USA). Two eluent-buffers were used to perform the chromatography. Buffer A is an aqueous solution containing 0.1 M triethylammonium acetate (TEAA), pH 7.0, and buffer B is an aqueous solution of 0.1 M TEAA, pH 7.0, containing 25% (v/v) acetonitrile. The whole procedure was undertaken at 50°C which is a non-denaturing temperature. Ion-pair RP-HPLC analysis was performed using the following linear gradient condition: flow rate 0.9 ml/min, 40–70% buffer B over 15 min. Peak quantification was performed by recording chromatograms at 260 nm and integrating peak areas.

### TLR3 activation assay with a reporter cell line

Human TLR3-expressing HEK293 cells (InvivoGen, San Diego, CA, USA) were cultured in Dulbecco’s modified Eagle medium (DMEM) supplemented with 4.5 g/l glucose, 2 mM L-glutamine, 10% heat-inactivated fetal bovine serum (FBS), 1% penicillin/streptomycin in the presence of blasticidin (Sigma-Aldrich, St Louis, MO, USA) (30 μg/ml), zeocin (InvivoGen) (100 μg/ml), and normocin (InvivoGen) (100 μg/ml). For stimulation, the cells were seeded on a 96-well culture plate at 5 × 10^4^ cells/well and incubated overnight at 37°C. The attached cells were treated with various concentrations of dsRNA, NVT, or poly (I:C) (InvivoGen) and CpG (InvivoGen) for 24 h, and 20 μl of supernatant and 180 μl of Quanti-Blue were reacted in a 96-well enzyme-linked immunosorbent assay (ELISA) plate. While incubating at 37°C for 1-3 h, the absorbance was measured at 655 nm when the most color development occurred.

### RNA extraction

C57BL/6 mice were injected intramuscularly with optimal dose (10 μg) of NVT, poly(I:C), or CpG (25). Five hours later, total RNA was isolated from the local iLN of the immunized mice using TRIzol reagent (Thermo Fisher Scientific). Briefly, the cells from iLN were lysed by treatment with 1 ml of TRIzol, and 0.2 ml of chloroform was added to the lysate. After centrifugation at 12,000 × *g* for 15 min at 4°C, the aqueous phase containing RNA was transferred to a new tube. Then, the same amount of isopropanol was added and incubated for 10 min at room temperature, followed by centrifugation for 10 min at 12,000 × *g* at 4°C. The RNA pellets were washed with 75% ethanol, air-dried, and resuspended in RNase-free water. The amount of total RNA was measured at 260 nm with NanoDrop (Molecular Devices, San Jose, CA, USA). The purity was confirmed at 260/280 nm (a ratio between 1.8 - 2.0 is considered pure) and 260/230 nm (a ratio between 2.0 - 2.2 is considered pure).

### Quantitative real-time PCR

One Step TB Green PrimeScript RT-PCR kit (Takara Bio, Shiga, Japan) was used according to the manufacturer’s specifications. Briefly, RNA samples were mixed in a 96-well reaction plate with 2× One Step TB Green RT-PCR Buffer 4, PrimeScript 1 step Enzyme Mix 2, forward and reverse primers (10 μM), and RNase-free dH_2_O. Then, PCR reactions were performed with pre-incubation at 42°C for 5 min, followed by 40 thermal cycles at 95°C for 10 s and 55°C for 30 s using a CFX real-time PCR system (Bio-Rad, Hercules, CA, USA). The expression level of target genes was normalized to the reference gene HPRT and calculated using the 2*^−ΔΔCt^* method as previously described with minor modifications (26). Primers were selected from previous study (27) and synthesized by Cosmogenetech (Seoul, Korea). The specific primer sequences are shown in **Table S2**.

### An accelerated stability test of NVT

To test NVT for long-term stability, the substance was stored in an accelerated condition at 25 ± 2°C and relative humidity of 60 ± 5% under the ICH guideline (https://www.ich.org/page/quality-guidelines) on “Stability testing of new drug substances and drug products”. Endotoxin test was performed using the Pierce™ LAL Chromogenic Endotoxin Quantitation Kit (Thermo Fisher Scientific, Waltham, MA, USA) according to the manufacturer’s instructions. Their appearance and concentration were determined for each period, and the stability was monitored by electrophoresis on an 1% agarose gel.

### Preclinical good laboratory practices toxicity profiling

Preclinical GLP toxicity profiling experiments of the NVT administered through intramuscular and subcutaneous routes were undertaken at a GLP-certified organization (Biotoxtech, Cheongju, Korea) according to the Organisation for Economic Co-operation and Development (OECD) protocol (https://www.oecd.org/chemicalsafety/testing/good-laboratory-practiceglp.htm). The test profiles include single dose, dose range finding test, repeated dose toxicity tests (two doses, four doses, and recovery test) for Sprague Dawley rats and New Zealand white rabbits, antigenicity assays [passive cutaneous anaphylaxis (PCA) and active systemic anaphylaxis (ASA)] for guinea pigs, and *in vivo* micronucleus assay for ICR mice. Also*, in vitro* chromosomal aberration assay using a Chinese hamster lung cell line, and *in vitro* bacterial reverse mutation test (Ames test) for *Salmonella typhimurium* and *Escherichia coli* were performed (**Table 1**).

**Table 1:** Summary of GLP toxicity study results for NVT.

Test	Route/ Animal	Regimen	Results
Single Dose Toxicity Test	SC/SD Rat	A single dosing of 0, 10, 20 or 40 mg/kg for 6-week-old male and female SD rat	No mortality was observed. The approximate lethal dose was judged to exceed 40 mg/kg for both genders.
Repeated Dose Toxicity Test: Two dose	SC/SD Rat	Once a week for 2 weeks 0, 4 or 16 mg/kg for 6-week-old SD rat	No mortality was observed. The high dose of the 4-week repeated dosing toxicity test should be set to less than 4 mg/kg for males and 16 mg/kg for females.
Repeated Dose Toxicity Test: Four dose and 2 weeks recovery	SC/SD Rat	Once a week for 4 weeks (for male 0, 0.75, 1.5 or 3mg/kg, and fore female 0, 1.5, 3 or 6 mg/kg) followed by 2 weeks recovery	No mortality was observed. no observed adverse effect level (NOAEL) and maximum tolerant dose (MTD) to be 3 mg/kg for male and 6 mg/kg for female.
Single Dose Toxicity Test	IM/SD Rat	A single dosing of 0.8, 1.6 or 3.2 mg/kg for 6-week-old male and female SD rat	No mortality and general symptoms were observed. The MTD and lethal dose should exceed 3.2 mg per rat head.
Repeated Dose Toxicity Test: Two dose	IM/SD Rat	Once a week for 2 weeks 0.4, 0.8 or 1.6 mg/kg for 6-week-old SD rat	No mortality and general symptoms were observed. The high dose of the repeated-dose toxicity test for more than 4 weeks should be set to 0.8 mg/animal.
Single Dose Toxicity Test	IM /NZW Rabbit	A single dosing of 8 mg per rabbit	No mortality occurred in both sexes. No abnormal changes were observed at autopsy. The MTD should exceed 8 mg per rabbit head.
Repeated Dose Toxicity Test: Two dose	IM /NZW Rabbit	Once a week for 2 weeks 2, 4 or 8 mg per rabbit	No mortality and general symptoms were observed. The high dose of the repeated-dose toxicity test for 4 weeks should be set at 8 mg/animal.
*In vivo* Micronucleus Assay	IM /ICR Mouse	Standard protocol of OECD TG 474: https://www.oecd-ilibrary.org. The NVT was intramuscular administrated at 1.6, 0.8, 0.4 or 0.2 mg per animal, twice in 24 h intervals to the ICR male mice to evaluate effect on the micronuclei induction on mouse bone marrow cells	NVT did not cause micronucleus induction for mouse bone marrow cells.
*In vitro* Chromosomal Aberration Assay	NA/ Chinese hamster lung cell line	Standard protocol of OECD TG 473; https://www.oecd-ilibrary.org/	The test substance NVT did not cause chromosomal abnormalities at 250, 125, 62.5, 31.3, 15.6, 7.81, 3.91 and 1.95 μg/ml.
Antigenicity assay (PCA, ASA)	ID and IV /Guinea pig	Standard protocol of PCA and ASA; https://www.fda.gov/media/72228/download	NVT was negative for both PCA and ASA and was judged to be non-antigenic.
Bacterial Reverse Mutation Test, Ames Test	NA/ *Salmonella* Typhimurium, *Escherichia coli*	Standard protocol of OECD TG 471; https://www.oecd-ilibrary.org/	At all doses of each strain regardless of the presence or absence of metabolic activation, the number of revertant mutant colonies did not exceed twice that of the negative control group while in the positive control group, the revertant colonies for each strain increased more than twice than that of the negative control group. Therefore, NVT is considered to be non-mutagenic.

PCA; Passive Cutaneous Anaphylaxis, ASA; Active Systemic Anaphylaxis,

SD Rat; Sprague Dawley Rat, NZW Rabbit; New Zealand White Rabbit,

IM; intramuscular, SC; subcutaneous, ID; intradermal, IV; intravenous, NA; not applicable.

### Analysis of innate immune response

C57BL/6 female mice (n=4 per group) were administered either with 2 μg of ovalbumin (OVA; InvivoGen) alone or formulated with 10 μg of NVT or10 μg of poly(I:C) as an adjuvant *via* the intramuscular route. At 0, 6 or 24 h, the draining iLN was harvested to dissociate into a single cell suspension. The cells were labeled with a fixable viability dye to distinguish live cells from dead cells followed by surface staining for macrophage (CD11b^+^F4/80^+^), neutrophil (CD11b^+^Ly6G^+^), and DCs (CD11c^hi^MHC-II^hi^) (**Supplementary Figure 1**). The specificity of staining was confirmed by fluorescence minus one and the migration of innate cells was expressed as an absolute number after flow cytometric analysis. To analyze the activation of DCs, CD11c^hi^MHC-II^hi^ cells were gated and then the intensity of the surface expression of CD40, CD80, CD86, and MHC class II was determined by NovoCyte flow cytometry (ACEA Biosciences, San Diego, CA, USA). IFN-β was measured by sandwich ELISA according to the manufacturer’s instructions (BioLegend, San Diego, CA, USA).

### Immunization

To explore the efficacy of the adjuvants, C57BL/6 mice (n=5 per group, female, 6-8 weeks old) were immunized intramuscularly twice at two-week intervals with 2 μg of OVA with either alone or adjuvanted with 10 μg of NVT or 10 μg of poly (I:C). No severe clinical signs including weight loss were observed after immunization. Two weeks later, blood was collected to determine OVA-specific antibodies and the spleen was isolated to analyze OVA-specific T cell responses.

### Determination of OVA-specific antibodies

The 96-well microplates were coated with either 2 μg/ml of goat anti-mouse IgG antibody for standard curve construction or 5 μg/ml of OVA for OVA-specific antibody titer overnight at 4°C and then non-specific sites were blocked with 1% skim milk. Serially diluted mouse IgG antibodies or serum samples were incubated for 2 h at room temperature. For the OVA-specific Ig isotypes, horseradish peroxidase-conjugated anti-mouse IgG-, IgG1-, and IgG2c-antibodies were used as secondary antibodies and developed by using 3,3′,5,5′-tetramethylbenzidine substrate solution. The reaction was stopped with 0.5 M HCl and the absorbance was measured at 450-590 nm using a Multiskan Sky microplate reader (Thermo Fisher Scientific). A standard curve using mouse IgG was generated and antibody titers of serum samples were calculated as arbitrary units relative to the standard curve.

### T cell re-stimulation and intracellular cytokine staining

Two weeks after the last vaccination, spleens were obtained from immunized mice and dissociated into single cells. To examine OVA-specific T cell responses, the cell suspensions were restimulated with 20 μg/ml of OVA peptides (OVA_257-264_ and OVA_323-339_) (28) and incubated overnight at 37°C. Then, 1 μg/ml of a protein transport inhibitor GolgiPlug (BD Bioscience, Franklin Lakes, NJ, USA) was added and further cultured for 4-6 h. After washing with phosphate-buffered saline (PBS), the cells were stained with a viability dye, anti-CD4, anti-CD8, and anti-CD44 antibodies for 20 min at 4°C. For intracellular staining, the cells were permeabilized and fixed using a Cytofix/Cytoperm solution (BD Bioscience) and stained with anti-IFN-γ and anti-IL-4 antibodies for 20 min at 4°C. The cells were washed and resuspended with FACS buffer (PBS containing 1% FBS and 0.1% NaN_3_) and analyzed by flow cytometry.

### Statistical analysis

Experimental results were expressed as the mean value ± standard deviation (S.D.). The Kruskal-Wallis test followed by Dunn’s post-test was used for multiple comparison of more than three groups by using GraphPad Prism version 6.00 for Windows (GraphPad Software, La Jolla, CA, USA, www.graphpad.com). *p<0.05 was considered statistically significant.

## Result

### Synthesis of dsRNA and selection of candidate lengths as a novel adjuvant

**Figure 1:**
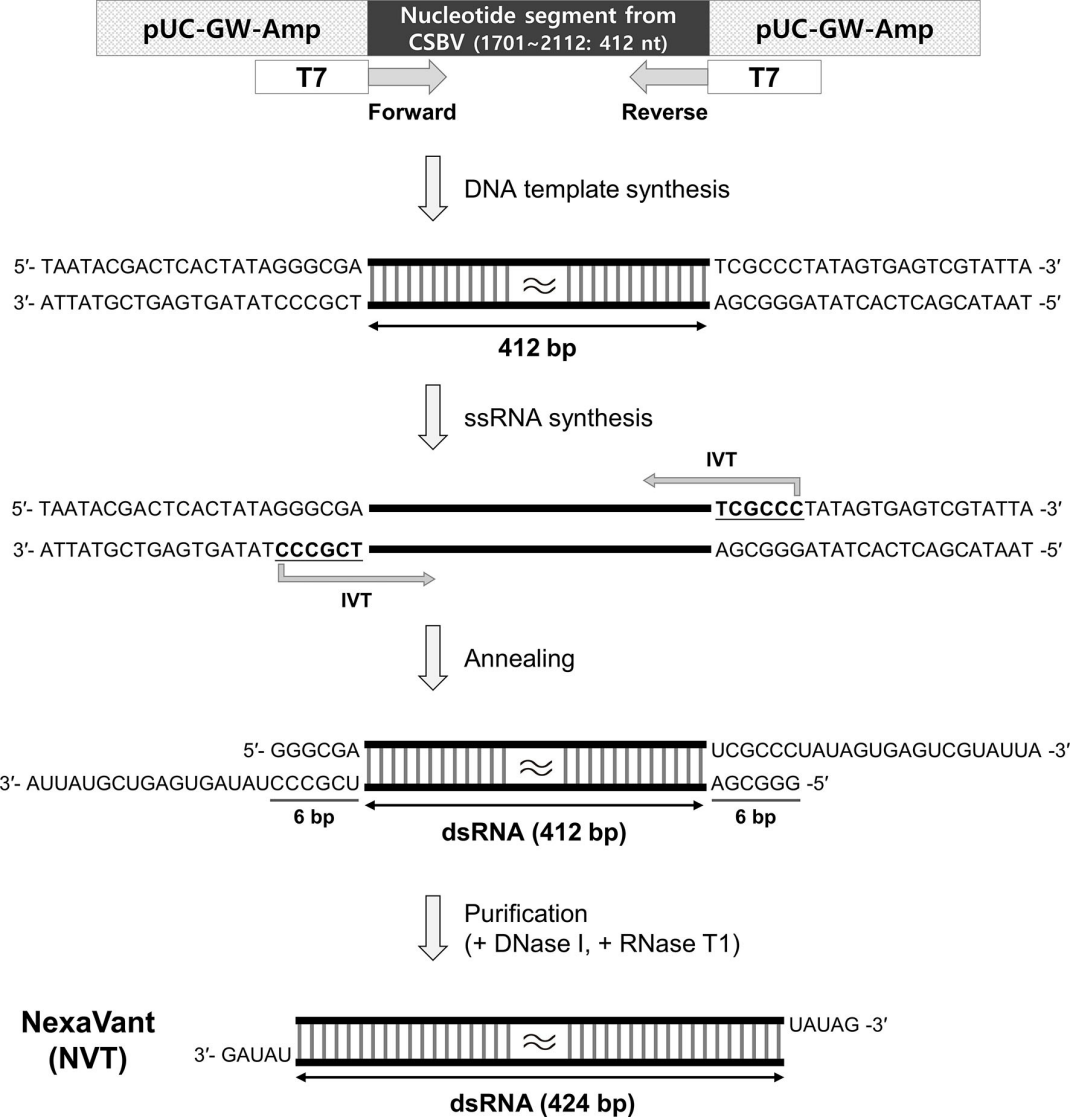
Schematic illustration of NexaVant (NVT) synthesis. The partial nucleotide segment (1701-2112; 412 nucleotides) from the CSBV genome was cloned into the vector. Forward and reverse PCR primers containing the T7 RNA promoter sequence were designed for the DNA template. After *in vitro* transcription (IVT) on the DNA template, complementary RNA strands were annealed to form dsRNA. Template DNAs and non-specific ssRNAs were removed with the treatment of DNase I and RNase T1 to generate a dsRNA structure having UAUAG-3′ at both ends and the final product was purified through column purification. nt, nucleotide.

The nucleotide segment (1,701-3,360; 1,660 nucleotides) from CSBV genome (GenBank accession number KF960044.1), which does not match with a human DNA sequence, was cloned into a vector. Then, PCR products of various sizes were produced by 3′-serial deletion and used to synthesize dsRNAs of 319, 397, 466, 508, 664, 733, 822, 885, 1,032, 1,153, or 1,648 bp length by IVT. When each dsRNA was electrophoresed on an 1% agarose gel, dsRNA longer than 508 bp showed polymeric impurities similar to poly(I:C), indicating the need to further select dsRNA of less than 500 bp (data not shown). Since dsRNA tend to activate TLR3 signaling, we tested whether the synthetic dsRNA could act as a TLR3 agonist. To this end, the TLR3-expressing reporter cell line was stimulated with dsRNA of 319 bp to 508 bp in length. The induction of TLR3 activation was similar regardless of the dsRNA size (**Supplementary Figure 2A**). To select a specific length of dsRNA that was the most effective as an adjuvant, mice were intramuscularly injected with OVA formulated with each of the dsRNAs twice at intervals of 2 weeks, and OVA-specific IgG levels in each serum were measured 2 weeks after boosting. Unlike the results of *in vitro* assays, dsRNAs shorter than 300 bp were found to be less effective in inducing antigen-specific antibodies as vaccine adjuvants *in vivo*, suggesting that candidate molecules were further selected from dsRNAs with lengths of 400 to 500 bp (**Supplementary Figure 2B**). Next, to determine the dsRNA size for optimal production efficiency, dsRNAs of various lengths were synthesized under the same conditions and their yields were compared on an 1% agarose gel. The yield of dsRNA synthesis was highest at 424 bp to 460 bp, about 120 μg per 20 μl reaction (**Supplementary Figure 2C**). In consideration of adjuvanticity, production efficiency, and manufacturing cost, dsRNA with a length of 424 bp (approximately 275 kDa) was selected as a final candidate for product analyses, and the partial genomic sequence (1,701-2,112; 412 nucleotides) of CSBV was used for cloning to synthesize it (**Figure 1**).

### Production of NVT

When dsRNA is synthesized with a commercial kit, ssRNA and DNA template are removed by treating the product with RNase A and DNase I. However, since RNase A can partially or completely degrade dsRNA (29), there is a possibility that it may affect the stability of the product after synthesis or purification. Considering that RNase T1 specifically degrades ssRNA at G residues and cannot cleave dsRNA, we used RNase T1 instead of RNase A for dsRNA purification. This step generated 424 bp dsRNA with UAUAG-3′ at both ends (**Figure 1**). We named this RNase T1-treated final product NexaVant (NVT). Treatment of NVT with RNase III alone or in combination with RNase T1 generated approximately 25 bp dsRNAs, indicating that NVT consists of dsRNA that forms a completely complementary pairing (**Supplementary Figure 3**). We also found the importance of RNase T1 use for the synthesis and purification of NVT by confirming that NVT is completely degraded by RNase A (**Supplementary Figure 3**).

### Long-term stability and physicochemical characteristics of NVT

NVT was tested for stability under accelerated storage conditions (25 ± 2°C). The result showed that the appearance, concentration, and molecular size of NVT were unaffected for 6 months (**Figure 2A** and **Table 2**). Next, an RP-HPLC assay was used to confirm that the finally-produced NVT is a single compound. The RP-HPLC study confirmed that NVT was eluted as a single peak at a retention time of 17.601 min and accordingly exhibited almost 100% purity, demonstrating that NVT exhibits an extreme homogeneity (**Figure 2B**). To determine whether NVT acts as a TLR3 ligand, human TLR3-expressing HEK 293 cells were stimulated with 10, 50, or 100 μg/ml of NVT, or 100 μg/ml of CpG (negative control), or poly(I:C) (positive control), and NF-κB/AP-1 activation was measured. NVT induced TLR3 activation in a dose-dependent manner *in vitro* (**Figure 2C**). To determine whether the expression of viral nucleic acid sensors *in vivo* is increased by NVT, mice were injected intramuscularly with 10 μg of NVT, CpG, or poly(I:C), then local draining iLN were isolated and the expression of TLR3, MDA5, and RIG-I was measured by RT-qPCR analysis. We found that NVT promoted the expression of MDA5 and RIG-I as well as TLR3 to the same level as poly(I:C) (**Supplementary Figure 4**). For biomedical product development, the *in vivo* kinetics of the candidate molecule is essential. Notably, pharmacokinetic data of poly(I:C) were difficult to obtain due to the lack of analytical methods for such a heterogeneous mixture (15). In contrast, RT-qPCR reactions can be used to determine the exact copy number of NVT in blood. Indeed, we were able to quantify the content of NVT in blood by collecting blood from rats injected subcutaneously with NVT, extracting RNA, and performing RT-qPCR. The pharmacokinetic analysis showed that the amount of NVT present in the blood decreased rapidly (**Figure 2D**), indicating that unpredictable responses by NVT caused by long-term retention in the body are unlikely.

**Table 2:** An accelerated stability test of NVT .

Test items	Pass criteria	#Duration of storage		
		0 month	3 months	6 months
Appearance	colorless transparent liquid	same as left	same as left	same as left
Concentration	≥3 mg/ml	3.3	3.5	3.4
A260/A280 ratio	1.8~2.2	2.1	2.1	2.1
Size on gel	A single distinct band about 424 bp (no visible two or smeared bands)	same as left	same as left	same as left
Endotoxin	<5 EU/ml	<5 EU/ml	<5 EU/ml	<5 EU/ml

#stored for indicated month at 25 ± 2°C, relative humidity 60 ± 5%.

**Figure 2:**
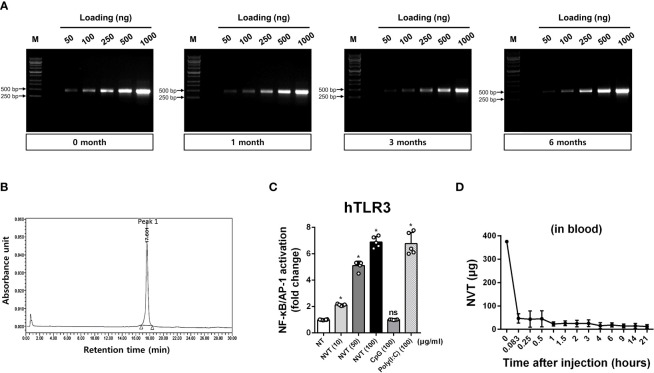
Stability and biological characteristics of NVT **(A)** NVT was stored at 25 ± 2°C for up to 6 months. NVT bands at various concentrations were identified by electrophoresis on 1% agarose gel in each month indicated. **(B)** Homogeneity of NVT samples was analyzed by RP-HPLC using a two-eluent buffer system. Peak quantification was performed by recording chromatograms at 260 nm and integrating peak areas. **(C)** Human TLR3-expressing HEK293 cells were stimulated with 10, 50, or 100 μg/ml of NVT, or 100 μg/ml of CpG (negative control) or poly(I:C) (positive control). After 24 h, the supernatant and Quanti-Blue solution were mixed and reacted at 37°C for 1-3 h. The change in absorbance was measured at 655 nm and the TLR3 activity of the samples was normalized relative to non-treated (NT) control. **(D)** Rats (n=6 per group) were subcutaneously injected with 375 μg of NVT. Blood was collected at each indicated time point, total RNA was extracted, and RT-qPCR for NVT was performed to determine *in vivo* pharmacokinetics of NVT. *, *P* < 0.05; ns: not significant.

### NVT promotes innate immune responses by inducing DC activation and innate immune cell migration

**Figure 3:**
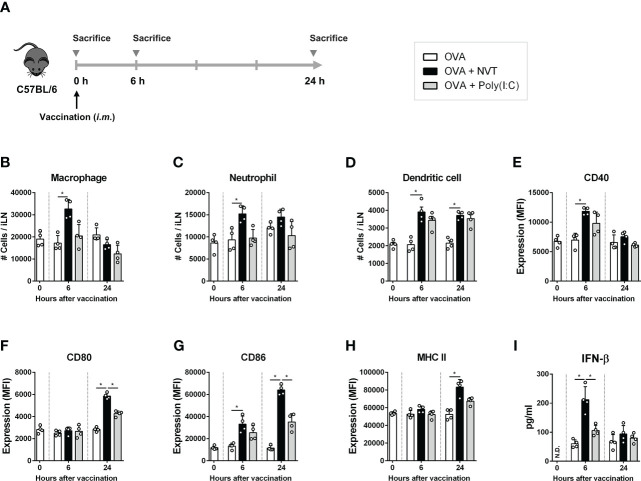
NVT prompts innate immune responses. **(A)** Experimental scheme. C57BL/6 female mice (n=4 per group) were injected into the thigh muscles of the right hind leg with OVA (2 μg) either alone or together with NVT (10 μg) or poly(I:C) (10 μg). At 0, 6 and 24 h, draining inguinal lymph node (iLN) on the right hind leg was collected, dissociated into single cells, and whole cells were counted. **(B-D)** Macrophages (CD11b^+^F4/80^+^), neutrophils (CD11b^+^Ly6G^+^) and DCs (CD11c^hi^MHC-II^hi^) were analyzed by flow cytometry, and the absolute numbers were calculated by multiplying the total iLN cells by their percentage. **(E-H)** The mean fluorescence intensity of CD40, CD80, CD86, and MHC-II expression on CD11c^hi^MHC-II^hi^ DCs was determined by flow cytometric analysis. **(I)** The concentration of IFN-β in serum was measured by ELISA. All data were expressed as the mean values ± S.D. *, *P* < 0.05; N.D., not detected.

To evaluate the role of NVT in the induction of an immune response as an adjuvant, C57BL/6 mice were intramuscularly injected with OVA either alone (control) or formulated with NVT or poly(I:C) as an adjuvant, and draining iLNs were collected 0, 6 and 24 h after injection to analyze innate immune cell migration and DC activation (**Figure 3A**). Compared to the data at 0 h, NVT efficiently promoted the migration of macrophages (CD11b^+^F4/80^+^) and neutrophils (CD11b^+^Ly6G^+^) as well as DCs (CD11c^hi^MHC-II^hi^) into draining iLN soon after the injection as poly(I:C) did (**Figures 3B-D**). Next, to measure the expression of DC maturation, the mean fluorescence intensity of CD40, CD80, CD86, and MHC-II in CD11c^hi^MHC-II^hi^ DCs was analyzed by flow cytometry, and we found both NVT and poly(I:C) promoted the activation of DCs. NVT significantly increased CD40 at 6 h compared to the control (OVA alone). After that, the expression of CD40 decreased and became similar to control at 24 h (**Figure 3E**). NVT enhanced the expression of the costimulatory molecules CD80 and CD86 at 24 h after injection, significantly higher than the control and poly(I:C) groups (**Figure 3F, G**). NVT also significantly promoted an increase in MHC class II expression compared to control at 24 h (**Figure 3H**). In addition to DC activation, NVT substantially promoted the production of IFN-β, the major effector cytokine induced by TLR3 agonists, compared to poly(I:C) at 6 h after injection, indicating that NVT provides strong *in vivo* activation of type I IFN signaling cascades (**Figure 3I**). Taken together, these results suggest NVT works as a potential adjuvant that can activate adaptive immunity through migration of APCs to LN, induction of DC activation, and promotion of IFN-β production.

### NVT enhances antigen-specific antibody response

**Figure 4:**
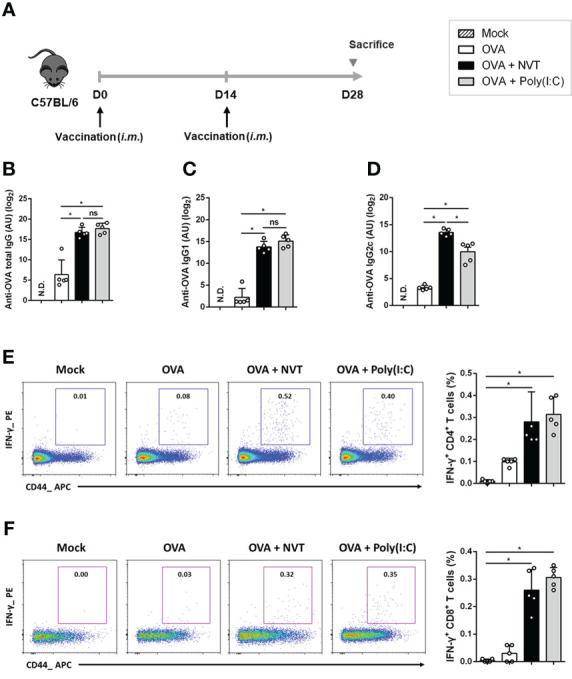
NVT potently enhances Th1-skewed antibody and T cell response. **(A)** C57BL/6 mice (n=5 per group) were primed and boosted *via* an intramuscular route with OVA (2 μg) either alone or adjuvanted with NVT (10 μg) or poly(I:C) (10 μg). **(B-D)** The levels of OVA-specific total IgG, IgG1, and IgG2c antibodies in the serum were measured at 2 weeks post-boosting. **(E, F)** Spleen cells were obtained from mice 2 weeks after boosting and restimulated with OVA peptides (OVA_257-264_ and OVA_323-339_). After blocking cytokine secretion with protein transport inhibitor, surface staining was performed with anti-CD4, anti-CD8, and anti-CD44 antibodies, followed by intracellular staining with anti-IFN-γ and anti-IL-4 antibodies. Flow cytometric analysis showed the proportion of IFN-γ^+^-expressing CD4 and CD8 T cells in the draining iLN. Data are presented as the mean values ± S.D. *, *P* < 0.05; ns, not significant; N.D., not detected.

Next, we examined the ability of NVT to induce antigen-specific antibody responses. C57BL/6 mice were immunized intramuscularly twice at two-week intervals with either OVA alone or adjuvanted with NVT or poly(I:C) according to the schedule (**Figure 4A**). Then, OVA-specific total IgG, IgG1, and IgG2c were measured in sera from mice two weeks after the last immunization. The groups in which OVA was formulated with an adjuvant such as NVT or poly(I:C) showed significant increases in antigen-specific IgG. Interestingly, NVT had higher levels of Th1-type IgG2c antibody but lower levels of Th2-type IgG1 antibody compared to poly(I:C) (**Figures 4B-D**). These results suggest that NVT is a strong effective vaccine adjuvant that can induce a potent Th1-biased antibody response.

### NVT induces antigen-specific Th1 and CD8^+^ T cell responses

To further explore whether NVT could promote the antigen-specific T cell response, C57BL/6 mice were vaccinated twice with either OVA alone or together with NVT or poly(I:C). Two weeks after boosting, splenocytes isolated from the mice were restimulated with epitopes of the OVA protein, OVA_257-264_ and OVA_323-339_, to analyze OVA-specific T cell responses (28). We found that OVA alone is insufficient to induce OVA-specific CD4^+^ T cell responses. NVT enhanced OVA-specific IFN-γ-producing CD4^+^ T cells to a level similar to that of poly(I:C) (**Figure 4E**) whereas OVA-specific IL-4-producing CD4^+^ T cells responses were not observed in any group (data not shown). Moreover, NVT promoted an OVA-specific cytotoxic T lymphocyte (CTL) response in the spleen to a level comparable to that of poly(I:C) (**Figure 4F**). Taken together, NVT would be an effective vaccine adjuvant that promotes potent Th1 and CTL responses, especially for vaccines against intracellular pathogens or cancers.

### GLP toxicity test of NVT

Pharmaceutical development includes GLP preclinical toxicology studies as required by regulatory agencies (30). Thus, we performed a GLP toxicity test to see if NVT is a safe substance without causing severe toxicity, and to obtain the clinical starting dose and safety margin we determined the maximum tolerated dose (MTD) and no observed adverse effect level (NOAEL). We first determined the MTD and lethal dose of NVT in rats, which is a standard toxicological test animal model (30), by subcutaneously administering doses of 0, 10, 20, and 40 mg/kg. No severe adverse effects were observed even at the highest dose and therefore lethal dose and MTD were considered as >40 mg/kg. In addition, when a repeated dose toxicity test (subcutaneous route in rats, 4 doses and recovery for 2 weeks) was performed, there was no mortality or serious symptoms, and NOAEL and MTD were confirmed to be 3 mg/kg for males and 6 mg/kg for females. Next, according to the standard protocol of the OECD, NVT (1.6, 0.8, 0.4, and 0.2 mg) was injected into ICR mice through the intramuscular route, and the effect on micronuclei induction was confirmed. There was no abnormality in the generation of micronuclear polychromatic red blood cells (MNPCE), indicating that NVT did not cause chromosomal damage, consistent with the *in vitro* chromosomal aberration assay. Lastly, the results of antigenicity (guinea pig) and Ames test (Salmonella) confirmed that NVT was a non-antigenic and non-mutagenic substance, respectively. Therefore, NVT is expected to be a safe vaccine adjuvant without serious toxicity in the range below the MTD and NOAEL (**Table 1**).

## Discussion

Although poly(I:C) has been a vaccine adjuvant candidate of interest for increasing the effectiveness of anti-viral and anti-cancer vaccines (22, 31), its toxicity problems and difficulties in quality-controlled manufacture and pharmacokinetic analysis due to its molecular nature (*i.e.*, mixed compounds with heterogeneous molecular structures) remain to be resolved for clinical application (15, 31). To overcome these problems, we developed a novel TLR3 agonist, NVT, and investigated its physicochemical properties, adjuvanticity, and safety. NVT displayed high purity, molecular homogeneity, measurable pharmacokinetics, long-term stability, and non-toxicity in various animals (**Table 1**). Mechanism studies demonstrated that NVT is effective for the migration of innate immune cells into local draining lymph node and the activation of DCs. Furthermore, NVT induced a Th1-biased antibody response superior to poly(I:C). Therefore, NVT would be a promising adjuvant for anti-viral or anti-cancer vaccines, successfully overcoming the problems of poly(I:C) as an adjuvant candidate.

To produce the intended effects an active pharmaceutical ingredient should possess consistent molecular characteristics, defined pharmacokinetics, and appropriate stability. The heterogeneous structure of poly(I:C) made exact pharmacokinetic analysis difficult and induced many unpredictable side effects in clinical trials (15). The current study demonstrated that NVT is a homogeneous molecule with a confirmed molecular structure that enables accurate pharmacokinetic analysis. Manufacturing of NVT is highly reproducible without sequence errors in PCR and IVT steps (data not shown), indicating that NVT can be mass-produced as an identical material with batch-to-batch consistency. Considering that the physical instability of the adjuvant to temperature changes can lead to loss of vaccine efficacy even within the cold chains (32), the long-term stability of NVT as determined by the accelerated stability test condition will be a huge advantage for commercial manufacturing, storage, and shipping of the final vaccine product.

NVT has excellent adjuvanticity in that it potently enhanced OVA-specific antibody responses; in particular, the production of IgG2c is significantly higher than poly(I:C). Notably, the production of IgG2c in C57BL/6 mice is associated with a Th1-type cellular immune response (33). Concomitantly with the antibody responses, NVT promoted the induction of antigen-specific Th1 and CTL responses rather than Th2-type responses. Since Th1-type immunity is essential for the removal of intracellular pathogens or tumors that are difficult to eliminate through antibody-mediated responses (34), NVT could be suitable as a vaccine adjuvant requiring cell-mediated responses for protection. Considering the possibility of antibody-dependent enhancement of infection and allergic reactions by Th2-biased vaccines (7, 35, 36), Th1-biased NVT could be selected for many vaccine applications.

We found that NVT could activate TLR3 and increase the expression of the viral RNA sensors MDA5 and RIG-I as much as poly(I:C) (**Supplementary Figure 4**). The increased expression of those viral RNA sensors may promote the production of type I IFNs and thereby potentiate anti-viral host defense by increasing recognition of and response to intracellular viral pathogens (37). Indeed, type I IFNs have broad biological effects on the defense against viral infection in the host (38). Type I IFNs are essential for DC maturation to promote Th1 cell differentiation (13). Type I IFNs also promote the formation of a germinal center (GC) and leads to selective amplification of the IgG2c^+^ GC B cells through Th1-dependent pathways (38). Consequently, type I IFNs augment IgG2c production with class switching (38, 39), and deficiency of IFNR results in a significant decrease in the production of Ag-specific IgG2a with an increase in IgG1 (40). These results imply that IFN-β induced by NVT would play a key role in the formation of GC B cells and the production of IgG2c as well as Th1 cellular immunity.

We demonstrated that NVT is a vaccine adjuvant that does not cause serious problems in GLP toxicity tests according to the OECD guidelines. Despite the potential of poly(I:C) as an effective vaccine adjuvant, its clinical application has been limited due to the possibility of various toxic side effects (22).. For instance, poly(I:C) can stimulate the production of factors that can exacerbate lupus nephritis in the nephritic kidney in a mouse animal model (41). Poly(I:C) can also cause abnormal expression of MHC class I molecules on beta islet cells and activate potentially autoreactive T cells in the pancreas, which can lead to autoimmune diseases such as diabetes in mice (42). In a human study, intravenous injection of poly(I:C) triggered toxicity such as fever, mild elevation of liver enzymes, and coagulation abnormalities (43). Moreover, a phase I clinical trial in advanced cancer reported that poly(I:C) complexes developed systemic allergic reactions in 2 of 32 patients (23). On the other hand, NVT is considered to be non-allergic because no inflammatory responses or abnormalities were observed in the PCA and ASA tests using the guinea pigs (**Table 1**). GLP toxicity results also showed that NVT does not cause mortality or severe problems in rats up to approximately 40 mg/kg *via* the subcutaneous route, and did not induce adverse effects in toxicity tests according to the OECD protocol in various animal models (**Table 1**). In addition, since NVT disappears rapidly after proper stimulation within the body, it is considered to be less likely to cause an abnormal response. A safety assessment of NVT in humans will be needed for future clinical applications.

Despite the excellent immunostimulatory potential of NVT, there is the possibility of non-delivery issue due to RNA degradation as RNA can be easily degraded by various physical or chemical factors (44). In particular, rapid degradation by enzymes such as ribonucleases *in vivo* may prevent delivery to APCs or lymphoid organs, limiting their role as immune stimulators (22). Poly(I:C) showed enhanced stability *in vivo* by combining poly-L-lysine and carboxymethyl cellulose (poly-ICLC) (45), implying that the stability of NVT can be further improved. Currently, many studies have demonstrated that combination of vaccines with emulsions or cationic liposomes not only protects the substance but also induces a more efficient immune response (46, 47). Thus, the formulation of the NVT with the chemicals or delivery system is expected to contribute to more effective vaccine efficacy as well as stability *in vivo*.

In conclusion, we demonstrated that NVT in the presence of an antigen successfully elicits antigen-specific T cell and antibody response. More importantly, NVT had no observed toxicity on administration to various animal models by various routes. In addition, NVT has a single molecular weight and defined structure and is feasible for mass production under quality-controlled manufacturing. Therefore, NVT could be a novel, effective, and safe adjuvant for human vaccines, especially for anti-viral or anti-cancer vaccines. In the future, optimized formulation of vaccine antigen and NVT is expected to increase antigen persistence and potentiate protective immune response.

## Data availability statement

The original contributions presented in the study are included in the article/**Supplementary Materials**. Further inquiries can be directed to the corresponding author.

## Ethics statement

The animal study was reviewed and approved by The Ethics Committee and Institutional Animal Care and Use Committee of the NA Vaccine Institute (Permit Number: NAVI-2019-0002).

## Author contributions

SH and KK designed this work. KK, SC, HB and CH carried out all experiments. KK, M-GL and SH analyzed and/or interpreted data. KK, SC, D-HK and SH prepared and reviewed the manuscript. All authors contributed to the article and approved the submitted version.

## References

[1] Di Pasquale A Preiss S Tavares Da Silva F Garcon N. Vaccine adjuvants: From 1920 to 2015 and beyond. Vaccines (Basel) (2015) 3(2):320–43. doi: 10.3390/vaccines3020320 PMC4494348 26343190

[2] Rapaka RR Cross AS McArthur MA. Using adjuvants to drive T cell responses for next-generation infectious disease vaccines. Vaccines (Basel) (2021) 9(8):820. doi: 10.3390/vaccines9080820 34451945 PMC8402546

[3] Pulendran B SA P O'Hagan DT. Emerging concepts in the science of vaccine adjuvants. Nat Rev Drug Discovery (2021) 20(6):454–75. doi: 10.1038/s41573-021-00163-y PMC8023785 33824489

[4] Awate S Babiuk LA Mutwiri G. Mechanisms of action of adjuvants. Front Immunol (2013) 4:114. doi: 10.3389/fimmu.2013.00114 23720661 PMC3655441

[5] Morrow MP Yan J Pankhong P Ferraro B Lewis MG Khan AS. Unique Th1/Th2 phenotypes induced during priming and memory phases by use of interleukin-12 (IL-12) or IL-28B vaccine adjuvants in rhesus macaques. Clin Vaccine Immunol (2010) 17(10):1493–9. doi: 10.1128/CVI.00181-10 PMC2952990 20685940

[6] Didierlaurent AM Morel S Lockman L Giannini SL Bisteau M Carlsen H. AS04, an aluminum salt- and TLR4 agonist-based adjuvant system, induces a transient localized innate immune response leading to enhanced adaptive immunity. J Immunol (2009) 183(10):6186–97. doi: 10.4049/jimmunol.0901474 19864596

[7] Tseng CT Sbrana E Iwata-Yoshikawa N Newman PC Garron T Atmar RL. Immunization with sars coronavirus vaccines leads to pulmonary immunopathology on challenge with the sars virus. PloS One (2012) 7(4):e35421. doi: 10.1371/journal.pone.0035421 22536382 PMC3335060

[8] Gin DY Slovin SF. Enhancing immunogenicity of cancer vaccines: QS-21 as an immune adjuvant. Curr Drug Ther (2011) 6(3):207–12. doi: 10.2174/157488511796391988 PMC4248601 25473385

[9] Bonam SR Partidos CD Halmuthur SKM Muller S. An overview of novel adjuvants designed for improving vaccine efficacy. Trends Pharmacol Sci (2017) 38(9):771–93. doi: 10.1016/j.tips.2017.06.002 28668223

[10] O'Hagan DT. MF59 is a safe and potent vaccine adjuvant that enhances protection against influenza virus infection. Expert Rev Vaccines (2007) 6(5):699–710. doi: 10.1586/14760584.6.5.699 17931151

[11] Wilkins AL Kazmin D Napolitani G Clutterbuck EA Pulendran B Siegrist CA. AS03- and MF59-adjuvanted influenza vaccines in children. Front Immunol (2017) 8:1760. doi: 10.3389/fimmu.2017.01760 29326687 PMC5733358

[12] Champion CR. Heplisav-b: A hepatitis b vaccine with a novel adjuvant. Ann Pharmacother (2021) 55(6):783–91. doi: 10.1177/1060028020962050 32988213

[13] Longhi MP Trumpfheller C Idoyaga J Caskey M Matos I Kluger C. Dendritic cells require a systemic type I interferon response to mature and induce CD4+ Th1 immunity with poly ic as adjuvant. J Exp Med (2009) 206(7):1589–602. doi: 10.1084/jem.20090247 PMC2715098 19564349

[14] Rouas R Lewalle P El Ouriaghli F Nowak B Duvillier H Martiat P. Poly(I:C) used for human dendritic cell maturation preserves their ability to secondarily secrete bioactive IL-12. Int Immunol (2004) 16(5):767–73. doi: 10.1093/intimm/dxh077 15096480

[15] Naumann K Wehner R Schwarze A Petzold C Schmitz M Rohayem J. Activation of dendritic cells by the novel toll-like receptor 3 agonist RGC100. Clin Dev Immunol (2013) 2013:283649. doi: 10.1155/2013/283649 24454470 PMC3878805

[16] Le Naour J Galluzzi L Zitvogel L Kroemer G Vacchelli E. Trial watch: TLR3 agonists in cancer therapy. Oncoimmunology (2020) 9(1):1771143. doi: 10.1080/2162402X.2020.1771143 32934877 PMC7466857

[17] Grunberg-Manago M Oritz PJ Ochoa S. Enzymatic synthesis of nucleic acidlike polynucleotides. Science (1955) 122(3176):907–10. doi: 10.1126/science.122.3176.907 13274047

[18] Machida H Kuninaka A Yoshino H. Relationship between the molecular size of poly I-poly c and its biological activity. Jpn J Microbiol (1976) 20(2):71–6. doi: 10.1111/j.1348-0421.1976.tb00911.x 948145

[19] Marchetti S Schellens JH. The impact of FDA and EMEA guidelines on drug development in relation to phase 0 trials. Br J Cancer (2007) 97(5):577–81. doi: 10.1038/sj.bjc.6603925 PMC2360360 17726450

[20] Nakano T Yamamura ET Fujita H Sone T Asano K. Novel methods for nucleotide length control in double-stranded polyinosinic-polycytidylic acid production using uneven length components. Biosci Biotechnol Biochem (2018) 82(11):1889–901. doi: 10.1080/09168451.2018.1501264 30079840

[21] Komal A Noreen M El-Kott AF. TLR3 agonists: RGC100, ARNAX, and poly-IC: A comparative review. Immunol Res (2021) 69(4):312–22. doi: 10.1007/s12026-021-09203-6 PMC8213534 34145551

[22] Hafner AM Corthesy B Merkle HP. Particulate formulations for the delivery of Poly(I:C) as vaccine adjuvant. Adv Drug Delivery Rev (2013) 65(10):1386–99. doi: 10.1016/j.addr.2013.05.013 23751781

[23] Krown SE Kerr D Stewart WE 2nd Field AK Oettgen HF. Phase I trials of Poly(I,C) complexes in advanced cancer. J Biol Response Mod (1985) 4(6):640–9. 2418162

[24] Mian MF Ahmed AN Rad M Babaian A Bowdish D Ashkar AA. Length of dsrna (Poly I:C) drives distinct innate immune responses, depending on the cell type. J Leukoc Biol (2013) 94(5):1025–36. doi: 10.1189/jlb.0312125 23911868

[25] Ahn SY Le CTT Ko EJ. Monophosphoryl lipid a and poly I:C combination adjuvant promoted ovalbumin-specific cell mediated immunity in mice model. Biol (Basel) (2021) 10(9):908. doi: 10.3390/biology10090908 PMC8471534 34571785

[26] Katragadda V Adem M Mohammad RA Sri Bhasyam S Battini K. Testosterone recuperates deteriorated Male fertility in cypermethrin intoxicated rats. Toxicol Res (2021) 37(1):125–34. doi: 10.1007/s43188-020-00046-1 PMC7806673 33489863

[27] Araya RE Jury J Bondar C Verdu EF Chirdo FG. Intraluminal administration of poly I:C causes an enteropathy that is exacerbated by administration of oral dietary antigen. PloS One (2014) 9(6):e99236. doi: 10.1371/journal.pone.0099236 24915573 PMC4051664

[28] Arnaboldi PM Roth-Walter F Mayer L. Suppression of Th1 and Th17, but not Th2, responses in a CD8(+) T cell-mediated model of oral tolerance. Mucosal Immunol (2009) 2(5):427–38. doi: 10.1038/mi.2009.93 PMC2857757 19571798

[29] Edy VG Szekely M Loviny T Dreyer C. Action of nucleases on double-stranded RNA. Eur J Biochem (1976) 61(2):563–72. doi: 10.1111/j.1432-1033.1976.tb10051.x 813998

[30] Piwoni K Jaeckel G Rasa A Alberts P. 4-week repeated dose rat glp toxicity study of oncolytic echo-7 virus rigvir administered intramuscularly with a 4-week recovery period. Toxicol Rep (2021) 8:230–8. doi: 10.1016/j.toxrep.2021.01.009 PMC7840795 33537211

[31] Qu J Hou Z Han Q Zhang C Tian Z Zhang J. Poly(I:C) exhibits an anti-cancer effect in human gastric adenocarcinoma cells which is dependent on RLRs. Int Immunopharmacol (2013) 17(3):814–20. doi: 10.1016/j.intimp.2013.08.013 24029594

[32] Darriba ML Cerutti ML Bruno L Cassataro J Pasquevich KA. Stability studies of the vaccine adjuvant U-Omp19. J Pharm Sci (2021) 110(2):707–18. doi: 10.1016/j.xphs.2020.10.011 PMC7815325 33058898

[33] Nazeri S Zakeri S Mehrizi AA Sardari S Djadid ND. Measuring of IgG2c isotype instead of IgG2a in immunized C57bl/6 mice with plasmodium vivax trap as a subunit vaccine candidate in order to correct interpretation of Th1 versus Th2 immune response. Exp Parasitol (2020) 216:107944. doi: 10.1016/j.exppara.2020.107944 32619431

[34] Knutson KL Disis ML. Tumor antigen-specific T helper cells in cancer immunity and immunotherapy. Cancer Immunol Immunother (2005) 54(8):721–8. doi: 10.1007/s00262-004-0653-2 PMC11032889 16010587

[35] Xu L Ma Z Li Y Pang Z Xiao S. Antibody dependent enhancement: Unavoidable problems in vaccine development. Adv Immunol (2021) 151:99–133. doi: 10.1016/bs.ai.2021.08.003 34656289 PMC8438590

[36] Ebenig A Muraleedharan S Kazmierski J Todt D Auste A Anzaghe M. Vaccine-associated enhanced respiratory pathology in covid-19 hamsters after T(H)2-biased immunization. Cell Rep (2022) 40(7):111214. doi: 10.1016/j.celrep.2022.111214 35952673 PMC9346010

[37] Banos-Lara Mdel R Ghosh A Guerrero-Plata A. Critical role of MDA5 in the interferon response induced by human metapneumovirus infection in dendritic cells and in vivo. J Virol (2013) 87(2):1242–51. doi: 10.1128/JVI.01213-12 PMC3554051 23152520

[38] Dahlgren MW Plumb AW Niss K Lahl K Brunak S Johansson-Lindbom B. Type I interferons promote germinal centers through b cell intrinsic signaling and dendritic cell dependent Th1 and tfh cell lineages. Front Immunol (2022) 13:932388. doi: 10.3389/fimmu.2022.932388 35911733 PMC9326081

[39] Swanson CL Wilson TJ Strauch P Colonna M Pelanda R Torres RM. Type I IFN enhances follicular b cell contribution to the T cell-independent antibody response. J Exp Med (2010) 207(7):1485–500. doi: 10.1084/jem.20092695 PMC2901065 20566717

[40] Coro ES Chang WL Baumgarth N. Type I IFN receptor signals directly stimulate local b cells early following influenza virus infection. J Immunol (2006) 176(7):4343–51. doi: 10.4049/jimmunol.176.7.4343 16547272

[41] Patole PS Grone HJ Segerer S Ciubar R Belemezova E Henger A. Viral double-stranded RNA aggravates lupus nephritis through toll-like receptor 3 on glomerular mesangial cells and antigen-presenting cells. J Am Soc Nephrol (2005) 16(5):1326–38. doi: 10.1681/ASN.2004100820 15772251

[42] Lang KS Recher M Junt T Navarini AA Harris NL Freigang S. Toll-like receptor engagement converts T-cell autoreactivity into overt autoimmune disease. Nat Med (2005) 11(2):138–45. doi: 10.1038/nm1176 15654326

[43] Freeman AI Al-Bussam N O'Malley JA Stutzman L Bjornsson S Carter WA. Pharmacologic effects of polyinosinic-polycytidylic acid in man. J Med Virol (1977) 1(2):79–93. doi: 10.1002/jmv.1890010202 347034

[44] Stahl-Hennig C Eisenblatter M Jasny E Rzehak T Tenner-Racz K Trumpfheller C. Synthetic double-stranded RNAs are adjuvants for the induction of T helper 1 and humoral immune responses to human papillomavirus in rhesus macaques. PloS Pathog (2009) 5(4):e1000373. doi: 10.1371/journal.ppat.1000373 19360120 PMC2660151

[45] Diaz-San Segundo F Dias CC Moraes MP Weiss M Perez-Martin E Salazar AM. Poly ICLC increases the potency of a replication-defective human adenovirus vectored foot-and-Mouth disease vaccine. Virology (2014) 468-470:283–92. doi: 10.1016/j.virol.2014.08.012 25216089

[46] Zaks K Jordan M Guth A Sellins K Kedl R Izzo A. Efficient immunization and cross-priming by vaccine adjuvants containing TLR3 or TLR9 agonists complexed to cationic liposomes. J Immunol (2006) 176(12):7335–45. doi: 10.4049/jimmunol.176.12.7335 16751377

[47] Nordly P Rose F Christensen D Nielsen HM Andersen P Agger EM. Immunity by formulation design: Induction of high CD8+ T-cell responses by Poly(I:C) incorporated into the CAF01 adjuvant *Via* a double emulsion method. J Control Release (2011) 150(3):307–17. doi: 10.1016/j.jconrel.2010.11.021 21111765

