# Supplementary material for: A novel defined TLR3 agonist as an effective vaccine adjuvant
Source: Front Immunol. 2023 Jan 24;14:1075291. doi: 10.3389/fimmu.2023.1075291 (PMC9902914; doi:10.3389/fimmu.2023.1075291)
Supplement: Supplementary file 1 [file Presentation_1.pptx]

## Slide 1
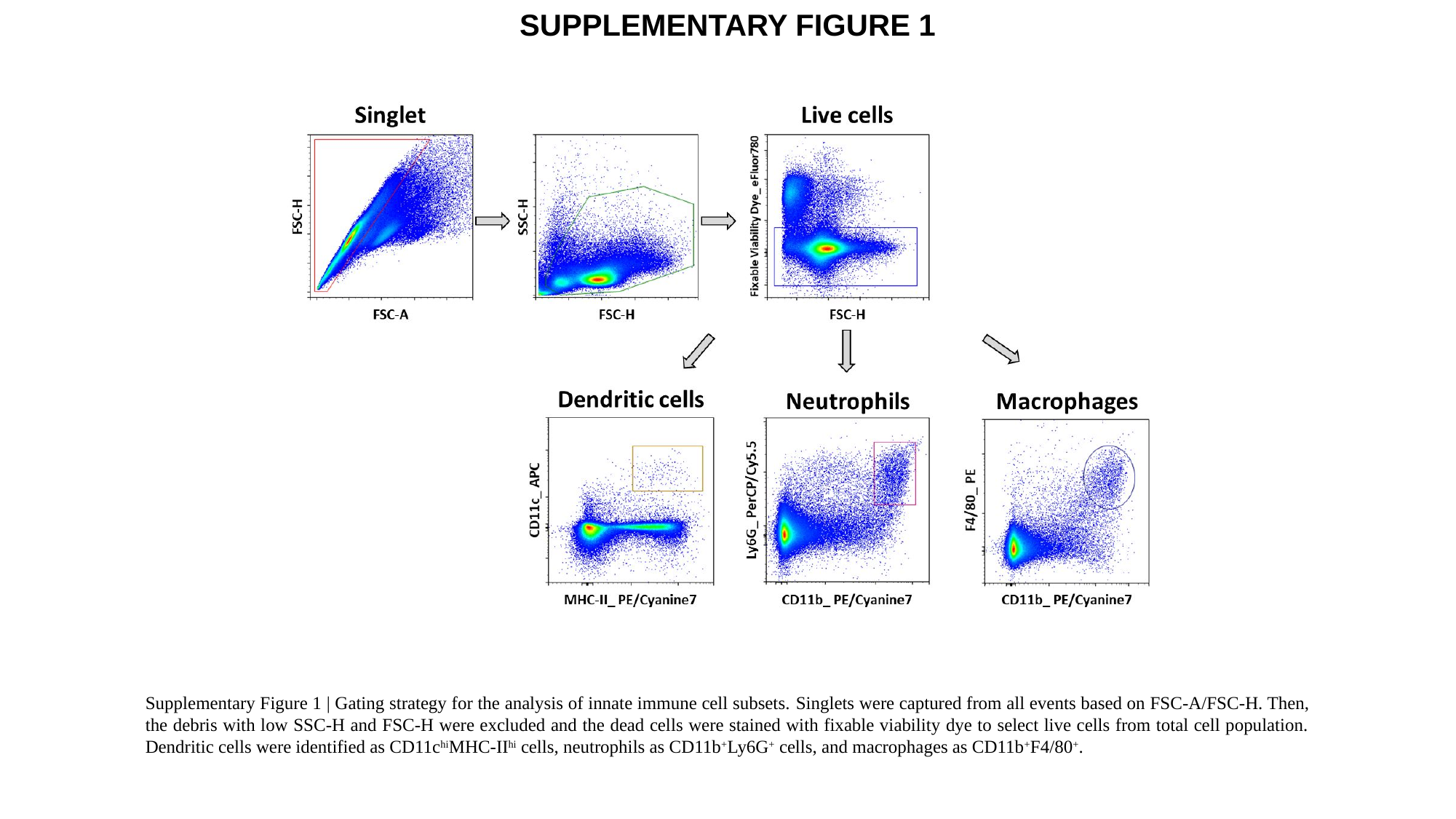

SUPPLEMENTARY FIGURE 1
Supplementary Figure 1 | Gating strategy for the analysis of innate immune cell subsets. Singlets were captured from all events based on FSC-A/FSC-H. Then, the debris with low SSC-H and FSC-H were excluded and the dead cells were stained with fixable viability dye to select live cells from total cell population. Dendritic cells were identified as CD11chiMHC-IIhi cells, neutrophils as CD11b+Ly6G+ cells, and macrophages as CD11b+F4/80+.

## Slide 2
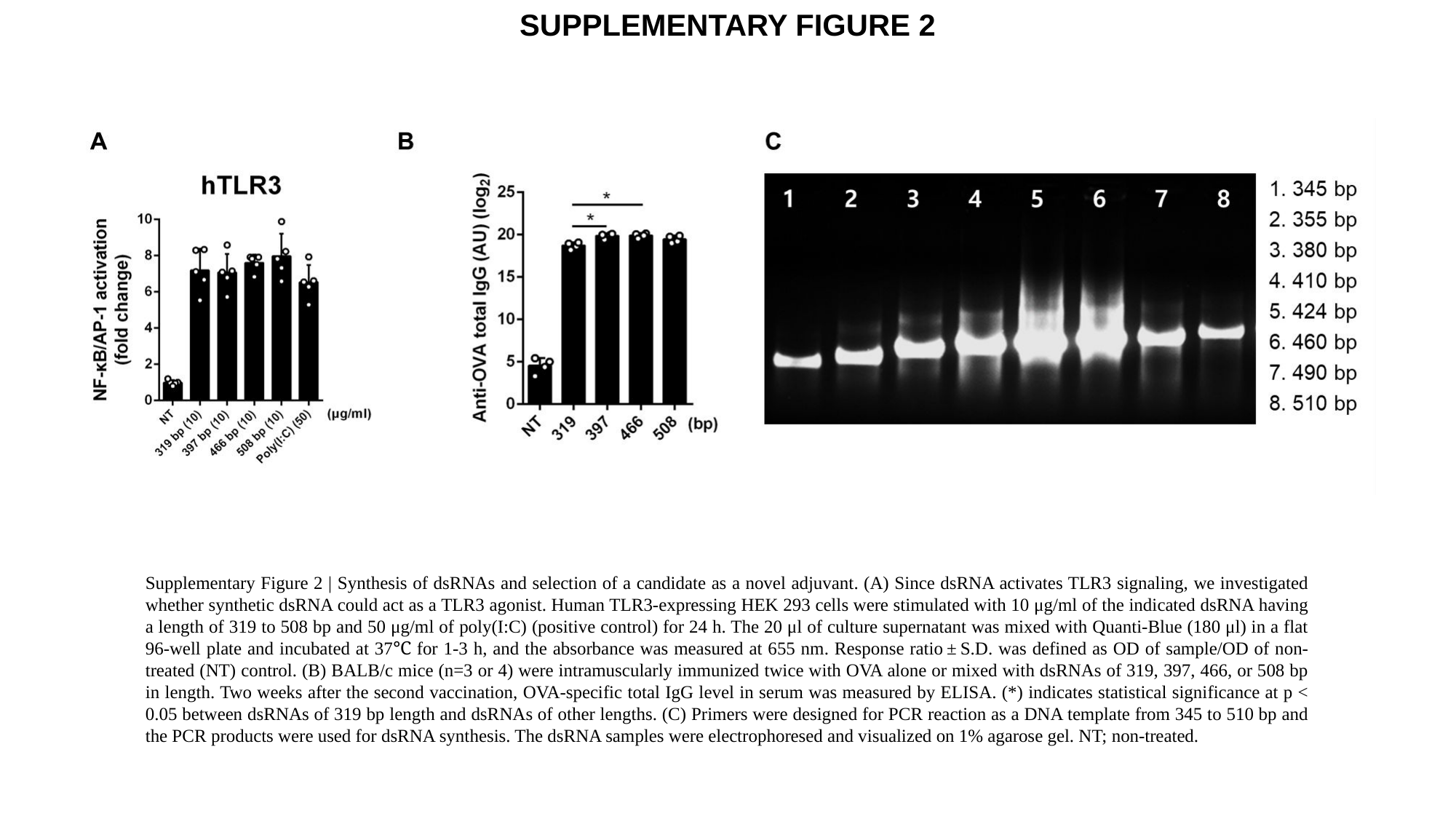

SUPPLEMENTARY FIGURE 2
Supplementary Figure 2 | Synthesis of dsRNAs and selection of a candidate as a novel adjuvant. (A) Since dsRNA activates TLR3 signaling, we investigated whether synthetic dsRNA could act as a TLR3 agonist. Human TLR3-expressing HEK 293 cells were stimulated with 10 μg/ml of the indicated dsRNA having a length of 319 to 508 bp and 50 μg/ml of poly(I:C) (positive control) for 24 h. The 20 μl of culture supernatant was mixed with Quanti-Blue (180 μl) in a flat 96-well plate and incubated at 37℃ for 1-3 h, and the absorbance was measured at 655 nm. Response ratio ± S.D. was defined as OD of sample/OD of non-treated (NT) control. (B) BALB/c mice (n=3 or 4) were intramuscularly immunized twice with OVA alone or mixed with dsRNAs of 319, 397, 466, or 508 bp in length. Two weeks after the second vaccination, OVA-specific total IgG level in serum was measured by ELISA. (*) indicates statistical significance at p < 0.05 between dsRNAs of 319 bp length and dsRNAs of other lengths. (C) Primers were designed for PCR reaction as a DNA template from 345 to 510 bp and the PCR products were used for dsRNA synthesis. The dsRNA samples were electrophoresed and visualized on 1% agarose gel. NT; non-treated.

## Slide 3
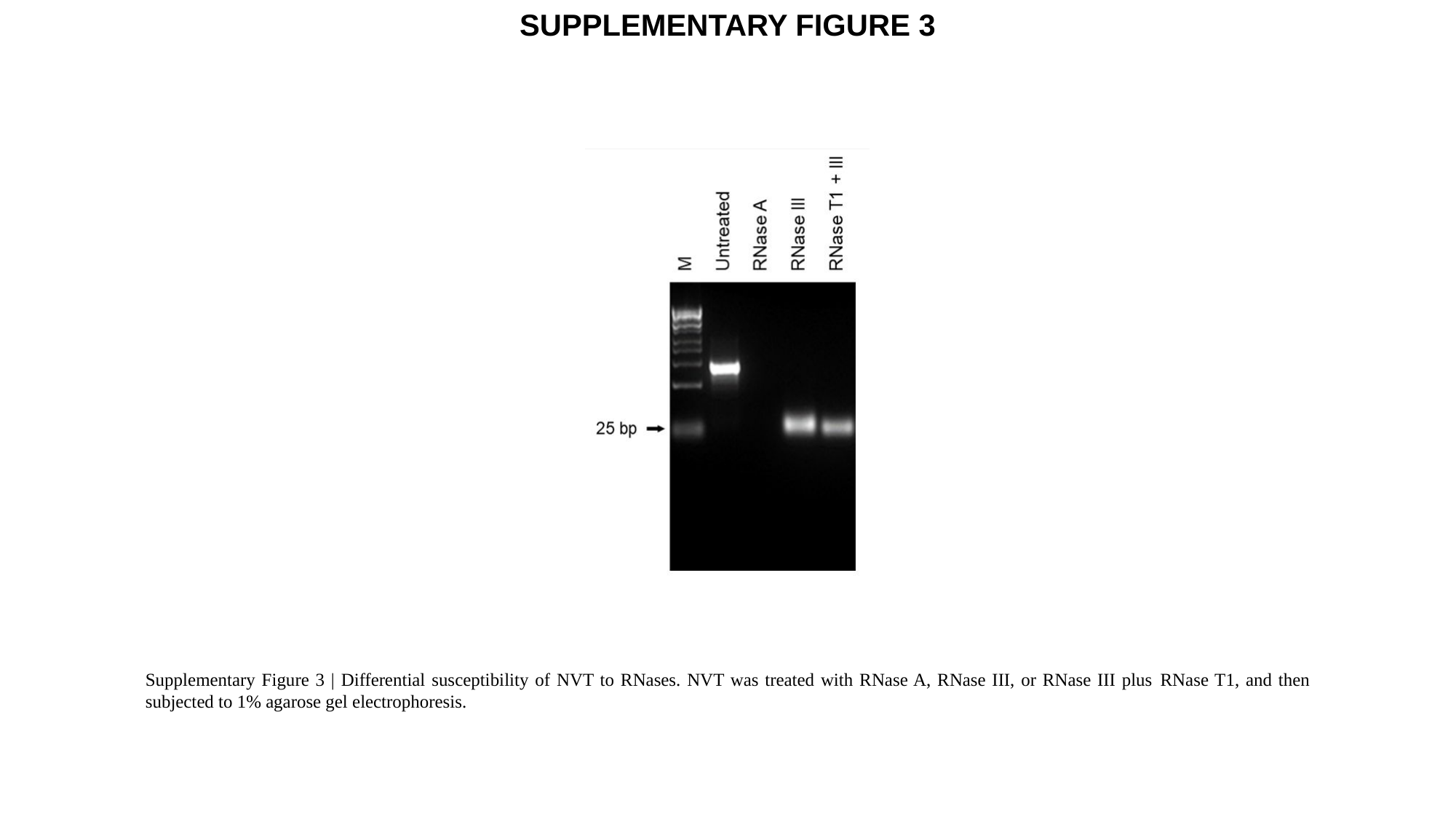

SUPPLEMENTARY FIGURE 3
Supplementary Figure 3 | Differential susceptibility of NVT to RNases. NVT was treated with RNase A, RNase III, or RNase III plus RNase T1, and then subjected to 1% agarose gel electrophoresis.

## Slide 4
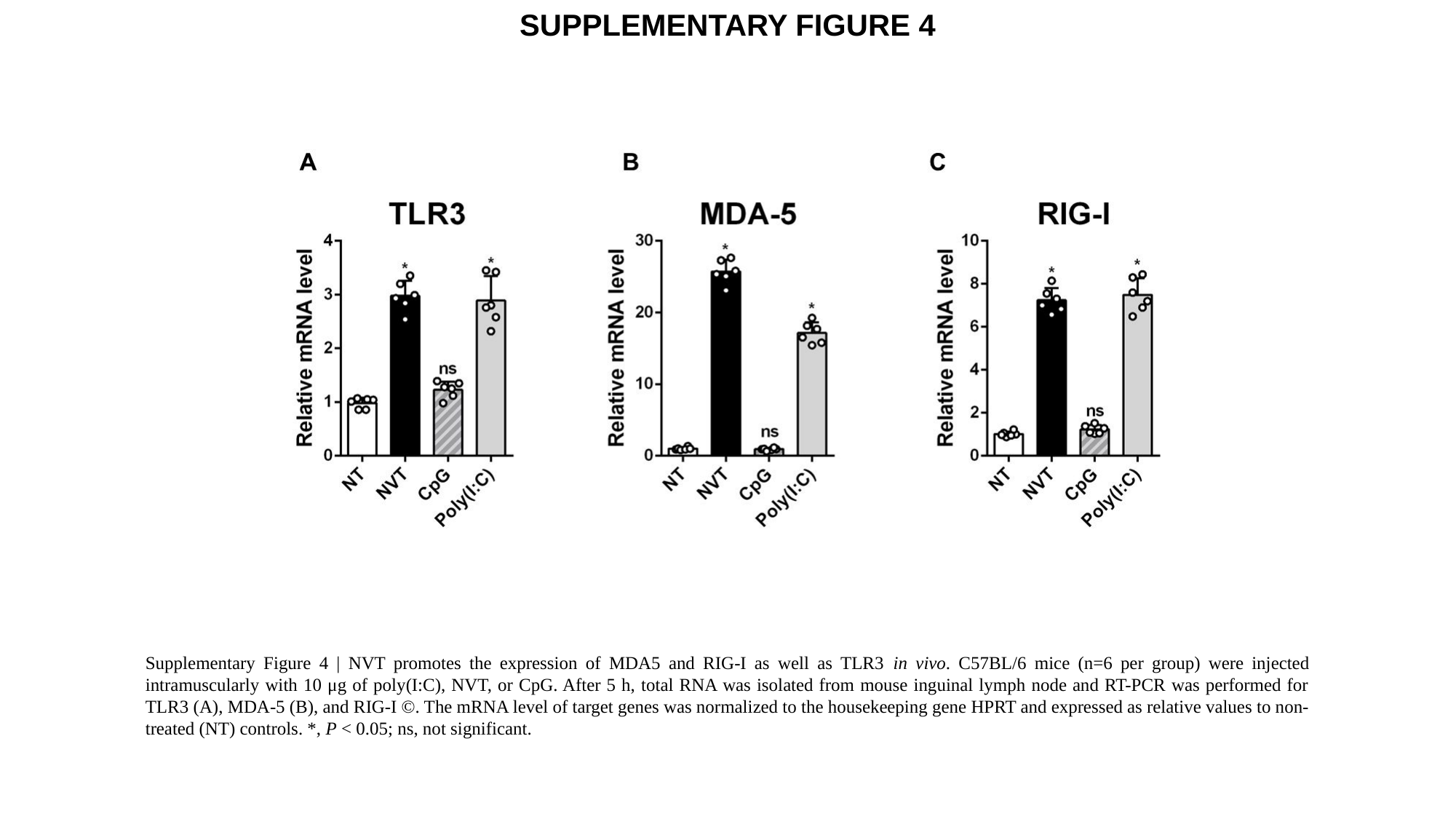

SUPPLEMENTARY FIGURE 4
Supplementary Figure 4 | NVT promotes the expression of MDA5 and RIG-I as well as TLR3 in vivo. C57BL/6 mice (n=6 per group) were injected intramuscularly with 10 μg of poly(I:C), NVT, or CpG. After 5 h, total RNA was isolated from mouse inguinal lymph node and RT-PCR was performed for TLR3 (A), MDA-5 (B), and RIG-I ©. The mRNA level of target genes was normalized to the housekeeping gene HPRT and expressed as relative values to non-treated (NT) controls. *, P < 0.05; ns, not significant.
