# Supplementary material for: A novel defined TLR3 agonist as an effective vaccine adjuvant
Source: Front Immunol. 2023 Jan 24;14:1075291. doi: 10.3389/fimmu.2023.1075291 (PMC9902914; doi:10.3389/fimmu.2023.1075291)
Supplement: Supplementary file 2 [file Table_1.docx]

Table S1. Mouse antibody list used in this study

| **Antibody** | **Fluorochrome** | **Clone** | **Purpose** | **Vendor** | **Cat no** |
| --- | --- | --- | --- | --- | --- |
| Viability dye | eFluor780 | - | Flow cytometry | eBioscience | 65-0865-14 |
| mCD11b | PE/Cyanine7 | M1/70 | Flow cytometry  (For innate cells) | BioLegend | 101216 |
| mCD11c | APC | N418 |  | BioLegend | 117310 |
| mLy6G | PerCP/Cyanine5.5 | 1A8 |  | BioLegend | 127616 |
| mF4/80 | PE | BM8 |  | BioLegend | 123110 |
| mCD40 | FITC | 3/23 | Flow cytometry  (For DC activation) | BioLegend | 124607 |
| mCD80 | PE | 16-10A1 |  | BioLegend | 104708 |
| mCD86 | PE/Cyanine5 | GL-1 |  | BioLegend | 105016 |
| mMHC II | PE/Cyanine7 | AF6-120.1 |  | BioLegend | 116420 |
| mCD4 | PE/Cyanine7 | RM4-5 | Flow cytometry  (For T cell response) | BioLegend | 100528 |
| mCD8a | FITC | 53-6.7 |  | BioLegend | 100706 |
| mCD44 | APC | IM7 |  | BioLegend | 103011 |
| mIFN-γ | PE | XMG1.2 |  | BioLegend | 505808 |
| mIL-4 | PerCP/Cyanine5.5 | 11B11 |  | BioLegend | 504123 |
| mIgM-HRP | - | - | ELISA | SouthernBiotech | 1021-05 |
| mIgG-HRP | - | - |  | SouthernBiotech | 1030-05 |
| mIgG1-HRP | - | - |  | SouthernBiotech | 1070-05 |
| mIgG2c-HRP | - | - |  | SouthernBiotech | 1030-05 |

| Table S2. Sequence information of PCR template and primers for NVT, and primers for qRT-PCR | |
| --- | --- |
| **Title** | **Sequence (5′-3′)** |
| Sequence for NVT template | Taatacagttttggactcaggtgtgagattttatgatcaggactatgaaggacaaataaccccaatggaatatgtaactgggttgtataacttttggtcagggccaatagagttacgttttgattttgtttcaaatgcgtttcacactggaacagtgattatatcagcggagtataatcgatcatctactaatacggatgagtgtcagtcacactcaacttatactaaaacgttccacttgggagaacaaaaatcagtacatttcactgtgccttatatatatgatactgttatgcggagaaatacggctagcgcctatttaccggtaactgattatgataaggcagataatgttagtagggcgcaggctacggggattagagcagaatctaaaatgagagtgaaagtgaga |
| Forward primer for NVT  Reverse primer for NVT | TAATACGACTCACTATAGGGCGATAaTACAGTTTTGGactcaggtgtgagatt  TAATACGACTCACTATAGGGCGATCTCACTTTCACTCTCATTTTAGATTCTGC |
| Forward primer for mouse TLR3  Reverse primer for mouse TLR3 | GAAGATGATGCAGTCTTTCCA  CCTGTATCATATTCTACTCCTTGC |
| Forward primer for mouse MDA-5  Reverse primer for mouse MDA-5 | CGATCCGAATGATTGATGCA  AGTTGGTCATTGCAACTGCT |
| Forward primer for mouse RIG-I  Reverse primer for mouse RIG-I | CAGACAGATCCGAGACACTA  TGCAAGACCTTTGGCCAGTT |
| Forward primer for mouse HPRT  Reverse primer for mouse HPRT | CAATGCAAACTTTGCTTTCC  CAAATCCAACAAAGTCTGGC |
